# Stevens: The Pose of the Body, the Type of the Cranium and the Visual Plane

**Published:** 1901-04

**Authors:** George T. Stevens

**Affiliations:** New York


### Correspondence.

STEVENS: THE POSE OF THE BODY, THE TYPE OF THE CRANIUM AND THE VISUAL PLANE.

*A CORRECTION.*

*Editor Buffalo Medical Journal:*

*Sir*—I observe that by some inadvertence the text and the illustrations in the abstract of my address before the Buffalo Academy of Medicine, reported in the JOURNAL, March, 1901, are badly mixed. As matters stand the cuts are quite misleading and confusing. I do not know that it is worth while to try to remedy the matter now, but I will venture to suggest the order in which the figures should have been placed, with explanations of the types intended to be represented:

In place of "Fig. 4," read Fig. 5, the tall head.

In place of "Fig. 5," read Fig. 6, the broad head.

In place of "Fig. 6," read Fig. 4, the long head.

Of course the cuts will remain out of their order, but that cannot be helped. The explanatory legends would, however, serve to help.

Of course I shall be glad to leave the matter to your judgment whether it will be best to attempt any correction and the manner of doing so if you think it best.

GEORGE T. STEVENS.

NEW YORK, March 10, 1901.

[The Editor regrets the error referred to by Dr. Stevens and is glad to present an authorised correction thereof, by publishing the distinguished author's letter.]

